# Assessing Fat Grafting in Breast Surgery: A Narrative Review of Evaluation Techniques

**DOI:** 10.3390/jcm13237209

**Published:** 2024-11-27

**Authors:** Razvan-George Bogdan, Alina Helgiu, Anca-Maria Cimpean, Cristian Ichim, Samuel Bogdan Todor, Mihai Iliescu-Glaja, Ioan Catalin Bodea, Zorin Petrisor Crainiceanu

**Affiliations:** 1 Plastic Surgery Department, “Victor Babes” University of Medicine and Pharmacy, 300041 Timisoara, Romania; razvan.bogdan@umft.ro (R.-G.B.); acimpeanu@umft.ro (A.-M.C.); mihai.iliescu.glaja@umft.ro (M.I.-G.); crainiceanu.zorin@umft.ro (Z.P.C.); 2 County Clinical Emergency Hospital Pius Branzeu, 300723 Timisoara, Romania; 3 Faculty of Medicine, “Lucian Blaga” University of Sibiu, 550024 Sibiu, Romania; alinahelgiu@yahoo.fr; 4 County Clinical Emergency Hospital of Sibiu, 550245 Sibiu, Romania; 5 General Surgery Department, “Iuliu Hatieganu” University of Medicine and Pharmacy, 400347 Cluj-Napoca, Romania; bodea_cata@yahoo.com; 6 Regional Institute of Gastroenterology and Hepatology Prof. Dr. Octavian Fodor, 400394 Cluj-Napoca, Romania

**Keywords:** fat grafting, breast surgery, breast augmentation

## Abstract

Fat grafting has gained prominence in reconstructive and aesthetic surgery, necessitating accurate assessment methods for evaluating graft volume retention. This paper reviews various techniques for assessing fat and fat grafts, including their benefits and limitations. Three-dimensional (3D) scanning offers highly accurate, non-invasive volumetric assessments with minimal interference from breathing patterns. Magnetic resonance imaging (MRI) is recognized as the gold standard, providing precise volumetric evaluations and sensitivity to complications like oil cysts and necrosis. Computed tomography (CT) is useful for fat volume assessment but may overestimate retention rates. Ultrasonography presents a reliable, non-invasive method for measuring subcutaneous fat thickness. Other methods, such as digital imaging, histological analysis, and weight estimation, contribute to fat graft quantification. The integration of these methodologies is essential for advancing fat graft assessment, promoting standardized practices, and improving patient outcomes in clinical settings.

## 1. Introduction

Adipose tissue has been used as a filler in plastic and cosmetic surgery since the late 1800s, with significant advancements in techniques over the past century. Coleman’s Lipostructure^®^, a refined procedure for adipocyte transplantation, has recently rekindled interest in this area [1]. Autologous fat grafts are an ideal solution for soft tissue defects, offering biocompatibility, cost-effectiveness, and multiple donor sites for fat extraction. The option for repeat grafting and the contouring benefits of liposuction make the procedure even more appealing [2]. The adipose transfer has evolved through three phases. Initially, the process involved surgical excision of fat before liposuction. The second phase followed liposuction, where fat was reinjected without preparation. The third phase, introduced by S.R. Coleman, focused on gentle purification methods that preserve fat tissue. Additionally, the discovery of multilineage stem cells in adipose tissue presents potential for autologous reconstruction, as these cells can differentiate into various types, including fat and bone [1]. 

Despite over a century passing since its inception, various challenges regarding fat grafts persist, with many questions remaining unanswered [2]. The main challenges of fat grafting include unpredictable outcomes and the need for fat growth and retention to maintain volume. Successful results depend on the density of viable cells and the rate of graft integration. To enhance fat graft longevity, it’s essential to stimulate neovascularization within the grafts [3]. Further research indicates that mature adipocytes are fragile and highly susceptible to trauma, hypoxia, and desiccation. In contrast, preadipocytes exhibit greater resistance to these conditions and tend to represent the largest surviving fraction of the graft after processing and implantation [4]. 

While the concept of autologous fat grafts for breast augmentation seems promising in principle, low graft durability has proven to be a persistent issue. Because of this, work on developing breast implants and several types of alternative filler materials is still being done. However, the 1987 guidelines established by the American Society of Plastic Surgeons, which limited procedures involving fat transfer to the breast, have seriously impeded progress in this area [2,5]. The regulation was lifted in 2007, leading to an acceleration in studies focusing on fat grafts [6]. The restriction was put in place because, with the imaging technology available at the time, it was challenging to differentiate between microcalcifications associated with breast cancer and those originating from fat grafts [5].

## 2. The Use of Fat Grafts in Breast Augmentation

While fat grafts can be used for many different purposes, such as enhancing the appearance of the face and breasts, they are most commonly utilized for these purposes [7,8]. Fat grafts are widely used in reconstructive surgeries, including breast reconstruction after cancer and congenital abnormalities such as Poland’s syndrome. It is also used in cosmetic breast augmentation for breast hypoplasia and tuberous breast deformities. Surgical technique and skill significantly affect outcomes, with graft loss rates varying from 20% to 90% in the first year. Three-dimensional photography tracking shows a 40% to 50% survival rate, with greater fat transfers leading to better persistence. Despite improvements in fat transfer methods, standardized protocols for fat collection, preparation, and injection are still lacking [9,10]. Many changes have been proposed for each step of the procedure, including donor site selection, graft harvesting and preparation, injection techniques, and postoperative care, in an effort to increase the survival rate of fat transplants. Two notable adaptations are the combined transfer approach that includes platelet-rich plasma and cell-assisted lipotransfer, which combines fat grafts with adipocyte stem cells [11,12]. Many studies have focused on fat graft transfer methods. Autologous fat grafting is done cautiously due to potential links with breast cancer. To approve the use of autologous fat grafting breasts, it is crucial to prove that fat grafts don’t increase cancer risk. Fat grafts in breast surgeries raise two oncological concerns: first, nodules and calcifications in the fat can mimic breast cancer during exams and imaging, delaying diagnosis. Second, adipocyte-derived aromatase or adipokines in fat grafts may promote local estrogen production, potentially aiding breast cancer development [13,14].

## 3. Composition and Properties of Human Adipose Tissue

The two primary adipocyte subtypes seen in animals are known to be white and brown adipocytes. Moreover, a third variety of adipocytes is known as beige adipocytes, which are closely linked to white adipocytes and exhibit traits of both brown and white adipocytes [15]. White fat stores energy and affects weight, while brown fat handles thermoregulation, though adults have little of it. Early 20th-century views saw fat tissue as thermo insulation and mechanical support unrelated to energy metabolism. Later research showed fat tissue plays a key role in energy regulation, controlled by neuronal and endocrine systems. Lafontan’s review details the evolution of fat tissue biology [16]. Adipocytes, responsible for storing fat, are just one part of white fat tissue. Surrounding them is the stroma, which includes cells such as adipose-derived stem cells (ADSCs), vascular endothelial cells, lymphocytes, macrophages, fibroblasts, and their precursors. Mature adipocytes are spherical and contain a single large fat droplet at their center [13,17,18].

Several methods are available to assess breast volume, though many don’t differentiate between adipose tissue and the mammary gland. These include MRI, CT scans, ultrasound, mammographic volume measurement, the Grossman-Roudner device, anthropometric measurements, the Archimedes (water displacement) method, casting, and others [19].

## 4. Imagistic Follow-Up After Fat Grafting

A critical concern regarding radiological changes is whether procedures such as breast reduction or fat grafting could be mistaken for malignancy. In a study comparing postoperative mammograms of patients who underwent reduction mammaplasty to those who had augmentation mammaplasty using the cell-assisted lipofilling (CAL) technique, the findings indicated that, unlike breast reduction, CAL did not introduce any new challenges for breast cancer surveillance [20]. 

Selecting the best method for radiological monitoring, as differentiating neoplastic from non-neoplastic breast lesions, can be difficult. Current research evaluates MRI, ultrasonography, and mammography. Parikh et al. introduced a classification system to better distinguish benign from malignant tumors on ultrasound [21]. When evaluating the efficacy of mammography, MRI, and ultrasonography, it was found that MRI was more effective at identifying problematic lesions, while ultrasonography was better at detecting benign abnormalities after fat grafting [22].

A different study examined preoperative and postoperative radiographic changes (measured at an average of 9 ± 5 months) to investigate the impact of fat grafting following breast-conserving surgery. In comparison to preoperative pictures, the results demonstrated a higher incidence of significant abnormalities in postoperative imaging, for example, scars, calcifications, and fat cysts [23].

The transition to 3D imaging technologies represented a major leap forward. Initial 3D imaging methods, like laser scanning and surface imaging, enabled non-invasive and accurate reconstruction of breast contours. However, these techniques struggled to capture the posterior breast border, which is essential for precise volume measurement [24,25].

Recent research has further advanced 3D imaging techniques, highlighting the combination of 3D imaging with centrifugation for fat processing. Studies reported varying fat retention rates and underscored the necessity for standardized measurement methods, with retention rates approximating 1/3 to 2/3 of cases depending on the technique employed [26,27].

## 5. Techniques for Assessing Adipose Tissue and Fat Grafts

At this time, there are many objective methods that can evaluate the fat volume. Measurements of distance are more accurate than those of volume using methods like magnetic resonance imaging (MRI), ultrasound, and computed tomography (CT). This restriction casts doubt on the precision of estimating fat retention rates with these techniques [28,29]. When it comes to measuring adipose tissue volumes, paraclinical investigations that use three-dimensional systems have higher rates of accuracy and reproducibility [30]. The availability of these technologies makes it simple to get a scan without hurting the body and create a virtual 3D model of the body. This could lessen variances and result in more consistent outcome ratings across many trials. Consequently, it makes sense to spread the word about this method’s clinical application for assessing fat transplant volume (Table 1).

## 6. Application of Magnetic Resonance Imaging (MRI) in Breast Tissue Evaluation

MRI is very useful for evaluating the breast after transplantation because of its great sensitivity in identifying problems after autologous fat transplantation, such as oil cysts, calcifications, or necrosis [41,42]. Magnetic resonance imaging (MRI) is becoming more and more utilized for breast imaging, and it is already considered the gold standard for detecting other breast disorders, such as implant rupture [42,43,44]. MRI can be used for quantitative evaluation in addition to qualitative judgments. For a long time, MRI-based volumetry has been utilized in other medical specialties, including neurosurgery, and its utilization in plastic surgery is also growing [32]. Notably, the first time MRI-based volumetry was used to assess volume after autologous fat transplantation to the breast was in 2010 [26].

MRI is likely the most precise method for noninvasively measuring tissue volume, making it the gold standard for breast volumetry [45].

Every subsequent volumetric assessment will be conducted using the boundaries that were set during the first volume assessment. It guarantees that the reference boundaries from the initial scan are precisely aligned with those in subsequent scans of the same patient, preserving consistency and comparability in the volume measurements. It does this by identifying the boundaries of the breast and their relationship to fixed bony landmarks [33].

The amount of fat in the retropectoral plane was assessed before surgery and one and six months postoperatively. Three-dimensional data was obtained by measuring the maximum anteroposterior projection, horizontal long axis, and vertical long axis, and these measurements were used to calculate the volume of transplanted fat via MRI scans. Fat survival was evaluated by determining the difference between preoperative and postoperative fat volumes. However, a limitation arose in accurately estimating grafted fat volume immediately after surgery, which may affect fat survival assessments [46].

Guidelines outlined by Herold et al. for volumetric assessment: the patients in this study were examined by MRI before the surgery and compared with another MRI postoperatively [26]. For each axial MRI slice, the breast area was calculated separately, with boundaries defined as follows: the skin represented the outermost boundary, and the posterior surface of the pectoralis muscle marked the inner boundary. The midpoint of the sternum served as the boundary towards the midline, and the lateral thoracic artery served as the boundary towards the side. Subsequently, a perpendicular line was drawn from the skin to the internal fascia of the pectoralis muscle. It used the OsiriX software (version 9.0.1) for volumetric calculations. 

A 1.5-Tesla MRI with a 4-channel breast coil was used to assess fat graft boundaries both pre- and postoperatively, with scans conducted three months after surgery based on a novel approach developed in previous research [33]. The reference MRI scan was taken the day after surgery when breast borders are at their widest, with the positioning related to the manubriosternal and midsagittal planes for identification. This reference scan was overlaid on subsequent breast scans to ensure consistency in measurements. The total retained volume was calculated using the formula:

[(V_3m_ − V_pre_)/V_injected_] × 100

where:

- V_3m_ represents the volume measured at three months after the intervention,
- V_pre_ is the initial volume recorded before the intervention, and
- V_injected_ refers to the total volume of the injected material.

The stratified volume change was determined using the formula:

[(V_3m_ − V_pre)_/(V_1post_ − V_pre_)] × 100

where:

- V_1post_ indicates the volume measured immediately after the intervention.

Additionally, the percent enhancement was calculated using:

[(V_3m_ − V_pre_)/V_pre_] × 100

which measures the percentage increase in volume relative to the initial volume before the intervention.

The delineation of periglandular fat and mammary gland margins was based on density and pixel values using Myrian software. Measurements were averaged across three assessments to ensure accuracy [28].

The measurement method involved using osseous markings to define the boundaries, with the anterior border delineated by the skin-air transition and the posterior border indicated by the ribs-pleura transition. The percentage of volume change caused by injected fat was calculated using the formula:

[(postoperativeBV − preoperativeBV)/transplantvolume] × 100%

At present, MRI volumetry is regarded as an exceptionally precise and reproducible method for volumetric evaluation, particularly in assessing volume retention following breast augmentation using autologous fat [33].

The total volume of transplanted adipose tissue was calculated using a radiological segmentation method, with cumulative net fat volumes derived by multiplying the infiltration volume by the fat percentage of the transplant, then compared to the retained volume in postoperative MRI images. The “percentage of gain” was assessed with the formula:

(postoperativeBV − preoperativeBV)/total transplanted fat volume × 100

where 100% retention indicates that the volume increase matches the transplant, and MRI volumetry of intrapectoral fat was conducted to determine the contribution of regional periglandular fat [29].

Systematic flaws in using MRI to measure breast volume changes have been identified, revealing that breast contour can alter after fat grafting. To minimize these errors, it is recommended that surgeons use fixed osseous markers as reference points for volumetric measurements. Additionally, breast shape can be affected by factors like posture and respiration, leading to variations in volume measurements and inconsistent retention rates, even with the same measurement tool [30,33].

Postoperative MRI images taken before significant graft resorption allow for accurate measurement of fat volume in the increased breast volume, with retention assessed through two scans—one preoperative and another after graft stabilization—using software that aligns the images with bone markers to ensure consistent analysis. The software defines skin boundaries and calculates breast volume increase, with the fat graft retention rate determined by dividing this increase by the initial fat volume injected [47].

The fat graft volume was overestimated by 6.28% with a standard deviation of 10.5% due to the method’s measuring error. 4.8% (interquartile range: 2.7–9.8) was the median interobserver variation, and 4.2% (interquartile range: 2.0–8.7) was the interobserver variation.

A 1.5-Tesla GE Signa HD (2008) with a customized mamma coil was used for the MRI assessment of fat-grafted breasts. T1-weighted Fast Spin Echo sequences were performed with a 512 × 512 matrix and 5 mm slice thickness in axial and coronal planes. The entire anterior chest wall was scanned one month before and four months after breast enlargement to measure fat graft volume changes. The manubriosternal joint served as a reference for aligning images using Osirix version 8.5 software. Regions of interest (ROIs) were marked based on specific anatomical landmarks. MRI scans also looked for new changes at the graft site, including T2-weighted short-tau inversion recovery sequences [48]. 

Several software applications are being used to determine the amount of fat based on MRI scans. For instance, the 32-bit free version of OsiriX software (Pixmeo, CA, Geneva, Switzerland) was used to determine the volume of the breasts [49]. O’Connell et al. are using Vectra XT three-dimensional imaging to assess symmetry in oncological breast reconstruction using a well-established protocol [50].

## 7. Dual-Energy X-Ray Absorptiometry (DEXA)

Dual-energy X-ray absorptiometry means that body fat can be quantified with accuracy (DEXA) [51]. DEXA accurately measures bone mineral content, non-bone lean mass, and fat mass across specific body regions, and recent software advancements enable it to assess visceral adipose tissue, making it the preferred method for tracking body composition changes over time due to its precision, affordability, and availability, particularly in evaluating obesity treatment outcomes [52].

## 8. Ultrasonography in Breast Tissue Assessment

Most of the studies on sonographic assessment after autologous fat transplantation mainly emphasize identifying complications rather than evaluating volume retention [26].

Recent advancements in ultrasonography for measuring breast fat post-lipofilling include the integration of shear wave elastography (SWE), which enhances elasticity assessments by providing quantitative data on tissue stiffness. A 2023 multicenter study demonstrated that combining 2D and 3D SWE with standard breast ultrasound significantly improved diagnostic accuracy, aiding in the differentiation of benign and malignant lesions [53].

An anchor point was placed along the anterior skin to maintain consistency in thickness measurements from ultrasound images during follow-up, with the retention rate of adipose tissue calculated using the formula:

1 − (T_6_ − T_0_)/(T_1_ − T_0_)

where T_0_, T_1_, and T_6_ represent baseline, one-month and six-month thickness measurements, respectively. While there was an observed increase in breast tissue thickness at four specific anchor points following fat grafting after implant removal for capsular contracture, the actual amount of adipose tissue retention was not quantified [37].

Overall, recent findings underscore the growing importance of ultrasonographic elastography in breast imaging, providing a trustworthy, non-invasive method for measuring breast adiposity and improving the management of breast lesions [38].

Ultrasound provides a dependable and straightforward method for measuring subcutaneous fat across various types of examinations, such as abdominal, gynecological, or superficial soft-tissue ultrasound [54]. It is recognized for its high accuracy, reproducibility, and sensitivity compared to alternative techniques, boasting a reliability rate exceeding 98% [55]. There is no need for any specific preparation before conducting subcutaneous fat measurements using ultrasound. Typically, between 40% and 60% of body fat accumulates subcutaneously [56]. Ultrasonography is frequently used to measure the thickness of the subcutaneous fat layer. For example, to obtain B-mode images, ultrasound examination using an 8-MHz linear array is employed, which is then analyzed to determine fat thickness at different points on the breast. This technique provides precise and non-invasive measurements, making it a standard in clinical evaluations [57].

## 9. Factors Influencing Volume Calculation

Variations in clinical aspects across studies, including volume measurement methods, fat processing techniques, injection session frequency, treatment indications, and follow-up duration, led to subgroup analysis, which revealed a significant difference between measurements from 3D scanning and CT (*p* = 0.01), suggesting CT may overestimate the retention rate [58].

Liu et al. discovered that variations in the respiratory state could impact breast volume measurements when using 3D scanning techniques and emphasized the importance of maintaining a consistent respiratory state for accurate volumetric changes [31]. The previous research also indicated that both respiration and the menstrual cycle can affect breast volume measurements [58,59]. Additionally, breast borders can vary among patients due to differences in breast shape and the posterior wall caused by thoracic cage movement. To minimize errors arising from different protocols, future criteria should include considerations of patients’ respiratory state, posture, menstrual cycle, and breast border.

Preoperative 3D breast imaging was performed using a JRCB-D scanner with ≤0.1 mm precision, following a validated protocol, and scans were aligned using “selection-based registration” to overlap the peribreast region. The volumetric change was calculated by creating a cylinder-like curve from the preoperative margin and determining the volume difference between the preoperative and postoperative cylinders [60].

Also, cyclical fluctuations when the mammal gland is still present change the volume of the breast. An MRI volumetric study outlined breast volume changes ranging from −5.5% to +8.1% due to menstrual cycle variations [29]. The preovulatory and postovulatory phases have also been studied, and there are some differences that should be taken into consideration [59]. Women who have previously given birth had a much greater overall retained fat volume compared to those without a history of lactation [60].

Another plausible explanation for the additional volume gain could be attributed to damage to adipocytes during fat transfer, resulting in a temporary reduction in volume. This volume reduction may then return to the original volume after a regeneration phase, which occurs sometime after the absorption of adipocytes, depending on the adipocyte life cycle [29].

Even to ensure precision in the calculation, Ueberreiter et al. considered BMI before and after surgery and made an association between BMI increment and breast volume augmentation. This factor must be taken into consideration; if the patient is losing weight, the breast volume is reduced (in patients with a BMI down by 9%, breast volume down by 20%), and vice versa [29].

It is important to note that a certain amount of postoperative swelling is anticipated when the second examination occurs (less than three hours after surgery), which may contribute to the observed 6.28% overestimation [47]. Other factors that are important in measuring the breast are even breathing during the procedure, posture, and breast shape, which can contribute to variability in measurements [58]. Moreover, the MRI technique scans the breast in a prone position, which may not align with the standard positioning used by plastic surgeons for inspection. This disparity in positioning can lead to measuring errors in breast volume assessment [30] (Table 2).

## 10. Conclusions

Magnetic Resonance Imaging (MRI) stands out as an accurate and repeatable method for assessing fat graft survival following autologous fat transplantation in breast augmentation, although it may face limitations in estimating grafted fat volume immediately post-surgery. With its high sensitivity and specificity, MRI remains the gold standard for breast volume assessment, allowing precise tissue differentiation without radiation exposure. However, its high cost and limited accessibility make it unsuitable for all patients, particularly those with certain implants. Computed Tomography (CT) and mammography provide rapid, high-resolution imaging but expose patients to radiation and offer limited differentiation of breast tissue types. Ultrasonography is a portable, real-time method but depends heavily on operator skill and is less effective for detailed fat measurement. While advanced methods like 3D imaging enhance accuracy, they are costly and less commonly used, and scanning can be affected by respiratory state and menstrual cycle, emphasizing the need for standardized protocols. Less invasive methods, such as bioelectrical impedance, are convenient but lack specificity for breast fat assessment. Each method presents unique advantages and limitations, and the choice depends on clinical needs, patient characteristics, and available resources.

## Figures and Tables

**Table 1:** Breast volume assessment techniques and features.

Method	Benefit	References
3D Scanning (JRCB-D Scanner)	Highly accurate (≤0.1 mm precision), non-invasive volumetric assessment, with minimized breathing effect during scans.	Liu et al. [31]
Magnetic Resonance Imaging (MRI)	Gold standard for precise, non-invasive volumetry, sensitive to complications (e.g., oil cysts, necrosis), consistent with fixed bone landmarks.	Herold et al. [26,32], Glovinski et al. [33]
Computed Tomography (CT)	Useful for volume assessment but may overestimate fat retention rates.	Basile et Basile [34]
Dual-energy X-ray absorptiometry (DEXA)	Accurate, cost-effective method for quantifying body fat and tracking composition changes.	Caan et al. [35] Iyengar et al. [36]
Ultrasonography	Reliable, non-invasive, high-precision tool for measuring subcutaneous fat thickness, sensitive for fat retention in breast imaging.	Sampathkumar et al. [37] Goddi [38]
Bioelectrical Impedance	Cost-effective, non-invasive estimation of body fat through tissue conductance/resistance differences.	Soguel et al. [39]
Excision and Caliper Measurement	Tissue biopsy followed by fat thickness measurements with caliper; highly precise, often repeated for accuracy.	Gentilucci et al. [40]

**Table 2:** Factors influencing Breast Volume Measurements.

Factors	Impact on Measurements
Measurement Technique	Different imaging methods (MRI, CT, ultrasound) yield varying results in volume accuracy and estimation.
Cyclical Fluctuations	Hormonal changes can cause breast volume variations, ranging from −5.5% to +8.1% during the menstrual cycle.
Parous Status	Women who have breastfed tend to retain more fat volume compared to those who haven’t.
Adipocyte Damage	Initial fat transfer can cause temporary volume loss, which may be compensated by tissue regeneration over time.
Body Mass Index (BMI)	Fluctuations in BMI can significantly impact breast volume; weight loss leads to decreased volume, while weight gain results in increased volume.
Postoperative Swelling	Immediate postoperative swelling can lead to an overestimation of breast volume in early assessments.
Breathing Patterns	Variations in breathing during imaging can lead to inconsistencies in volume measurements.
Posture	The patient’s posture during imaging affects measurement accuracy and consistency.
Breast Shape and Contour	Individual anatomical variations can lead to differences in measured breast volume across patients.
MRI Positioning	The prone positioning used in MRI may not correspond with the standard inspection positions used in clinical evaluations, potentially introducing measurement errors.
Timing of Assessments	The timing of MRI scans post-surgery affects accuracy-scans may overestimate retained fat due to swelling.

## Data Availability

Not applicable.

## References

[1] Mojallal A., Foyatier J.-L. (2004). Historical review of the use of adipose tissue transfer in plastic and reconstructive surgery. Ann. Chir. Plast. Esthét..

[2] Bayram Y., Sezgic M., Karakol P., Bozkurt M., Filinte G.T. (2019). The Use of Autologous Fat Grafts in Breast Surgery: A Literature Review. Arch. Plast. Surg..

[3] Trojahn Kølle S.-F., Oliveri R.S., Glovinski P.V., Elberg J.J., Fischer-Nielsen A., Drzewiecki K.T. (2012). Importance of Mesenchymal Stem Cells in Autologous Fat Grafting: A Systematic Review of Existing Studies. J. Plast. Surg. Hand Surg..

[4] Von Heimburg D., Hemmrich K., Haydarlioglu S., Staiger H., Pallua N. (2004). Comparison of Viable Cell Yield from Excised versus Aspirated Adipose Tissue. Cells Tissues Organs.

[5] Khouri R.K., Eisenmann-Klein M., Cardoso E., Cooley B.C., Kacher D., Gombos E., Baker T.J. (2012). Brava and Autologous Fat Transfer Is a Safe and Effective Breast Augmentation Alternative: Results of a 6-Year, 81-Patient, Prospective Multicenter Study. Plast. Reconstr. Surg..

[6] Gutowski K.A. (2009). Current Applications and Safety of Autologous Fat Grafts: A Report of the ASPS Fat Graft Task Force. Plast. Reconstr. Surg..

[7] Wang G., Ren Y., Cao W., Yang Y., Li S. (2013). Liposculpture and Fat Grafting for Aesthetic Correction of the Gluteal Concave Deformity Associated with Multiple Intragluteal Injection of Penicillin in Childhood. Aesthetic Plast. Surg..

[8] Rusciani Scorza A., Rusciani Scorza L., Troccola A., Micci D.M., Rauso R., Curinga G. (2012). Autologous Fat Transfer for Face Rejuvenation with Tumescent Technique Fat Harvesting and Saline Washing: A Report of 215 Cases. Dermatology.

[9] Choi M., Small K., Levovitz C., Lee C., Fadl A., Karp N.S. (2013). The Volumetric Analysis of Fat Graft Survival in Breast Reconstruction: *Plast*. Reconstr. Surg..

[10] Rosing J.H., Wong G., Wong M.S., Sahar D., Stevenson T.R., Pu L.L.Q. (2011). Autologous Fat Grafting for Primary Breast Augmentation: A Systematic Review. Aesthetic Plast. Surg..

[11] Yoshimura K., Sato K., Aoi N., Kurita M., Hirohi T., Harii K. (2008). Cell-Assisted Lipotransfer for Cosmetic Breast Augmentation: Supportive Use of Adipose-Derived Stem/Stromal Cells. Aesthetic Plast. Surg..

[12] Gentile P., Di Pasquali C., Bocchini I., Floris M., Eleonora T., Fiaschetti V., Floris R., Cervelli V. (2013). Breast Reconstruction with Autologous Fat Graft Mixed with Platelet-Rich Plasma. Surg. Innov..

[13] Wang Y.-Y., Lehuédé C., Laurent V., Dirat B., Dauvillier S., Bochet L., Le Gonidec S., Escourrou G., Valet P., Muller C. (2012). Adipose Tissue and Breast Epithelial Cells: A Dangerous Dynamic Duo in Breast Cancer. Cancer Lett..

[14] Pearl R.A., Leedham S.J., Pacifico M.D. (2012). The Safety of Autologous Fat Transfer in Breast Cancer: Lessons from Stem Cell Biology. J. Plast. Reconstr. Aesthet. Surg..

[15] Wu J., Boström P., Sparks L.M., Ye L., Choi J.H., Giang A.-H., Khandekar M., Virtanen K.A., Nuutila P., Schaart G. (2012). Beige Adipocytes Are a Distinct Type of Thermogenic Fat Cell in Mouse and Human. Cell.

[16] Lafontan M. (2012). Historical Perspectives in Fat Cell Biology: The Fat Cell as a Model for the Investigation of Hormonal and Metabolic Pathways. Am. J. Physiol.-Cell Physiol..

[17] Ouchi N., Parker J.L., Lugus J.J., Walsh K. (2011). Adipokines in Inflammation and Metabolic Disease. Nat. Rev. Immunol..

[18] Lindroos B., Suuronen R., Miettinen S. (2011). The Potential of Adipose Stem Cells in Regenerative Medicine. Stem Cell Rev. Rep..

[19] Kayar R., Civelek S., Cobanoglu M., Gungor O., Catal H., Emiroglu M. (2011). Five Methods of Breast Volume Measurement: A Comparative Study of Measurements of Specimen Volume in 30 Mastectomy Cases. Breast Cancer Basic Clin. Res..

[20] Rubin J.P., Coon D., Zuley M., Toy J., Asano Y., Kurita M., Aoi N., Harii K., Yoshimura K. (2012). Mammographic Changes after Fat Transfer to the Breast Compared with Changes after Breast Reduction: A Blinded Study. Plast. Reconstr. Surg..

[21] Parikh R.P., Doren E.L., Mooney B., Sun W.V., Laronga C., Smith P.D. (2012). Differentiating Fat Necrosis from Recurrent Malignancy in Fat-Grafted Breasts: An Imaging Classification System to Guide Management. Plast. Reconstr. Surg..

[22] Costantini M., Cipriani A., Belli P., Bufi E., Fubelli R., Visconti G., Salgarello M., Bonomo L. (2013). Radiological Findings in Mammary Autologous Fat Injections: A Multi-Technique Evaluation. Clin. Radiol..

[23] Juhl A.A., Redsted S., Engberg Damsgaard T. (2018). Autologous Fat Grafting after Breast Conserving Surgery: Breast Imaging Changes and Patient-Reported Outcome. J. Plast. Reconstr. Aesthet. Surg..

[24] Kovacs L., Eder M., Hollweck R., Zimmermann A., Settles M., Schneider A., Endlich M., Mueller A., Schwenzer-Zimmerer K., Papadopulos N.A. (2007). Comparison between Breast Volume Measurement Using 3D Surface Imaging and Classical Techniques. Breast.

[25] Kovacs L., Eder M., Hollweck R., Zimmermann A., Settles M., Schneider A., Udosic K., Schwenzer-Zimmerer K., Papadopulos N.A., Biemer E. (2006). New Aspects of Breast Volume Measurement Using 3-Dimensional Surface Imaging. Ann. Plast. Surg..

[26] Herold C., Ueberreiter K., Busche M.N., Vogt P.M. (2013). Autologous Fat Transplantation: Volumetric Tools for Estimation of Volume Survival. A Systematic Review. Aesthetic Plast. Surg..

[27] Petridou E., Kibiro M., Gladwell C., Malcolm P., Toms A., Juette A., Borga M., Dahlqvist Leinhard O., Romu T., Kasmai B. (2017). Breast Fat Volume Measurement Using Wide-Bore 3 T MRI: Comparison of Traditional Mammographic Density Evaluation with MRI Density Measurements Using Automatic Segmentation. Clin. Radiol..

[28] Khoobehi K. (2019). Commentary on: Identification of the Optimal Recipient Layer for Transplanted Fat: A Prospective Study on Breast Lipoaugmentation. Aesthet. Surg. J..

[29] Ueberreiter C.S., Ueberreiter K., Mohrmann C., Herm J., Herold C. (2021). Langzeitevaluation nach autologer Fetttransplantation zur Brustvergrößerung. Handchir. · Mikrochir. · Plast. Chir..

[30] Wang C., Luan J. (2018). Magnetic Resonance Imaging Versus 3-Dimensional Laser Scanning for Breast Volume Assessment After Breast Reconstruction. Ann. Plast. Surg..

[31] Liu C., Ji K., Sun J., Luan J. (2014). Does Respiration Influence Breast Volumetric Change Measurement with the Three-Dimensional Scanning Technique?. Aesthetic Plast. Surg..

[32] Herold C., Knobloch K., Stieglitz L.H., Samii A., Vogt P.M. (2010). Magnetic Resonance Imaging–Based Breast Volumetry in Breast Surgery: A Transfer from Neurosurgery. Plast. Reconstr. Surg..

[33] Glovinski P.V., Herly M., Müller F.C., Elberg J.J., Kølle S.-F.T., Fischer-Nielsen A., Thomsen C., Drzewiecki K.T. (2016). Avoiding a Systematic Error in Assessing Fat Graft Survival in the Breast with Repeated Magnetic Resonance Imaging. Plast. Reconstr. Surg. Glob. Open.

[34] Basile F.V., Basile A.R. (2017). Prospective Controlled Study of Chin Augmentation by Means of Fat Grafting. Plast. Reconstr. Surg..

[35] Caan B.J., Cespedes Feliciano E.M., Prado C.M., Alexeeff S., Kroenke C.H., Bradshaw P., Quesenberry C.P., Weltzien E.K., Castillo A.L., Olobatuyi T.A. (2018). Association of Muscle and Adiposity Measured by Computed Tomography with Survival in Patients with Nonmetastatic Breast Cancer. JAMA Oncol..

[36] Iyengar N.M., Arthur R., Manson J.E., Chlebowski R.T., Kroenke C.H., Peterson L., Cheng T.-Y.D., Feliciano E.C., Lane D., Luo J. (2019). Association of Body Fat and Risk of Breast Cancer in Postmenopausal Women with Normal Body Mass Index: A Secondary Analysis of a Randomized Clinical Trial and Observational Study. JAMA Oncol..

[37] Sampathkumar U., Nowroozilarki Z., Bordes M.C., Reece G.P., Hanson S.E., Markey M.K., Merchant F.A. (2021). Review of Quantitative Imaging for Objective Assessment of Fat Grafting Outcomes in Breast Surgery. Aesthet. Surg. J..

[38] Goddi A., Bonardi M., Alessi S. (2012). Breast Elastography: A Literature Review. J. Ultrasound.

[39] Soguel L., Durocher F., Tchernof A., Diorio C. (2017). Adiposity, Breast Density, and Breast Cancer Risk: Epidemiological and Biological Considerations. Eur. J. Cancer Prev..

[40] Gentilucci M., Mazzocchi M., Alfano C. (2020). Effects of Prophylactic Lipofilling After Radiotherapy Compared to Non–Fat Injected Breasts: A Randomized, Objective Study. Aesthet. Surg. J..

[41] Hyakusoku H., Ogawa R., Ono S., Ishii N., Hirakawa K. (2009). Complications after Autologous Fat Injection to the Breast. Plast. Reconstr. Surg..

[42] Zheng D.-N., Li Q.-F., Lei H., Zheng S.-W., Xie Y.-Z., Xu Q.-H., Yun X., Pu L.L.Q. (2008). Autologous Fat Grafting to the Breast for Cosmetic Enhancement: Experience in 66 Patients with Long-Term Follow Up. J. Plast. Reconstr. Aesthet. Surg..

[43] Bartella L., Smith C.S., Dershaw D.D., Liberman L. (2007). Imaging Breast Cancer. Radiol. Clin. North Am..

[44] DeMartini W., Lehman C., Partridge S. (2008). Breast MRI for Cancer Detection and Characterization. Acad. Radiol..

[45] Herly M., Müller F.C., Ørholt M., Hansen J., Sværke S., Hemmingsen M.N., Rasmussen B.S., Elberg J.J., Drzewiecki K.T., Vester-Glowinski P.V. (2019). The Current Gold Standard Breast Volumetry Technique Seems to Overestimate Fat Graft Volume Retention in the Breast: A Validation Study. J. Plast. Reconstr. Aesthet. Surg..

[46] Guimarães P.A.M.P., Sabino Neto M., Lage F.C., Guirado F.F., De Mello G.G.N., Ferreira L.M. (2019). Evaluation of Retropectoral Fat Grafting in Breast Reduction by Magnetic Resonance Imaging: A Pilot Study. Aesthet. Surg. J..

[47] Herly M., Ørholt M., Müller F.C., Hemmingsen M.N., Hansen J., Larsen A., Rasmussen B.S., Elberg J.J., Von Buchwald C., Drzewiecki K.T. (2020). New Validated Method for Measuring Fat Graft Retention in the Breast with MRI. Plast. Reconstr. Surg. Glob. Open.

[48] Kølle S.-F.T., Duscher D., Taudorf M., Fischer-Nielsen A., Svalgaard J.D., Munthe-Fog L., Jønsson B., Selvig P.B., Mamsen F.P., Katz A.J. (2020). Ex Vivo-Expanded Autologous Adipose Tissue-Derived Stromal Cells Ensure Enhanced Fat Graft Retention in Breast Augmentation: A Randomized Controlled Clinical Trial. Stem Cells Transl. Med..

[49] Gentile P., Kothari A., Casella D., Calabrese C. (2020). Fat Graft Enhanced with Adipose-Derived Stem Cells in Aesthetic Breast Augmentation: Clinical, Histological, and Instrumental Evaluation. Aesthet. Surg. J..

[50] O’Connell R.L., Khabra K., Bamber J.C., deSouza N., Meybodi F., Barry P.A., Rusby J.E. (2018). Validation of the Vectra XT Three-Dimensional Imaging System for Measuring Breast Volume and Symmetry Following Oncological Reconstruction. Breast Cancer Res. Treat..

[51] Petak S., Barbu C.G., Yu E.W., Fielding R., Mulligan K., Sabowitz B., Wu C.-H., Shepherd J.A. (2013). The Official Positions of the International Society for Clinical Densitometry: Body Composition Analysis Reporting. J. Clin. Densitom..

[52] Ponti F., Plazzi A., Guglielmi G., Marchesini G., Bazzocchi A. (2019). Body Composition, Dual-Energy X-Ray Absorptiometry and Obesity: The Paradigm of Fat (Re)Distribution. BJR Case Rep..

[53] Xu J., Zhang L., Wen W., He Y., Wei T., Zheng Y., Pan X., Li Y., Wu Y., Dong F. (2023). Evaluation of Standard Breast Ultrasonography by Adding Two-Dimensional and Three-Dimensional Shear Wave Elastography: A Prospective, Multicenter Trial. Eur. Radiol..

[54] Störchle P., Müller W., Sengeis M., Ahammer H., Fürhapter-Rieger A., Bachl N., Lackner S., Mörkl S., Holasek S. (2017). Standardized Ultrasound Measurement of Subcutaneous Fat Patterning: High Reliability and Accuracy in Groups Ranging from Lean to Obese. Ultrasound Med. Biol..

[55] Smith-Ryan A.E., Fultz S.N., Melvin M.N., Wingfield H.L., Woessner M.N. (2014). Reproducibility and Validity of A-Mode Ultrasound for Body Composition Measurement and Classification in Overweight and Obese Men and Women. PLoS ONE.

[56] Carpéné C., Les F., Hasnaoui M., Biron S., Marceau P., Richard D., Galitzky J., Joanisse D.R., Mauriège P. (2016). Anatomical Distribution of Primary Amine Oxidase Activity in Four Adipose Depots and Plasma of Severely Obese Women with or without a Dysmetabolic Profile. J. Physiol. Biochem..

[57] Calabrese S., Zingaretti N., De Francesco F., Riccio M., De Biasio F., Massarut S., Almesberger D., Parodi P.C. (2020). Long-Term Impact of Lipofilling in Hybrid Breast Reconstruction: Retrospective Analysis of Two Cohorts. Eur. J. Plast. Surg..

[58] Wang C., Liu C., Giatsidis G., Cheng H., Chen L., Kang D., Panayi A.C., Luan J. (2019). The Effect of Respiration on Breast Measurement Using Three-Dimensional Breast Imaging. Aesthetic Plast. Surg..

[59] Wang C., Luan J., Cheng H., Chen L., Li Z., Panayi A.C., Liu C. (2019). Menstrual Cycle-Related Fluctuations in Breast Volume Measured Using Three-Dimensional Imaging: Implications for Volumetric Evaluation in Breast Augmentation. Aesthetic Plast. Surg..

[60] Wang K., Mu D., Zhang X., Lin Y. (2021). Lactation History Affects Postoperative Fat Volume Retention Rate in Autologous Fat Grafting Breast Augmentation. Aesthetic Plast. Surg..

